# Generating opposition to universal health care policies in the United States: An analysis of private health industry advertising on Meta platforms

**DOI:** 10.1371/journal.pgph.0003244

**Published:** 2025-07-16

**Authors:** Kendra Chow, Marco Zenone, Nora Kenworthy, Beza Merid, Nason Maani

**Affiliations:** 1 Faculty of Public Health and Policy, London School of Hygiene and Tropical Medicine, London, United Kingdom; 2 School of Public Policy and Global Affairs, University of British Columbia, Vancouver, Canada; 3 Health Law Institute, Faculty of Law, University of Alberta, Edmonton, Canada; 4 School of Nursing & Health Studies, University of Washington Bothell, Bothell, Washington, United States of America; 5 School for the Future of Innovation in Society, Arizona State University, Tempe, Arizona, United States of America; 6 Global Health Policy Unit, The University of Edinburgh, Edinburgh, United Kingdom; PLOS: Public Library of Science, UNITED STATES OF AMERICA

## Abstract

In 2019, the Partnership for America’s Health Care Future (PAHCF), a private health industry lobby group, launched a campaign across Meta platforms (Facebook, Instagram) to generate opposition to universal health care policies in the United States. This study investigates the content and themes prevalent in PAHCF’s campaign and how these might shape public discourse and perceptions around universal health care policies. Using qualitative content analysis, 1675 advertisements were examined on Meta platforms within PAHCF’s campaign. Inductive methodology was applied to develop a coding framework. Details of campaign spend and number of impressions advertisements received were also collected. The qualitative coding strategies identified three overarching campaign foci: policy targets, claims and themes, and targeted appeal groups. These elements were found to strategically and mutually reinforce one another to generate the narrative that proposed universal health care policies will be detrimental to public health, the economy, and society. Analysis identified that PAHCF engages in strategies common among unhealthy commodity industries. Social media in this instance powerfully perpetuated PAHCF messages that undermined universal health care efforts and contributed to the commercial determinants of health impacts of this industry. These findings indicate that the private health care industry is participating in wider commercial determinants of health activities, acting to protect their profits to the detriment of public health. Like other campaigns by unhealthy commodity industries, PAHCF’s campaign is designed to increase doubt in the benefits of health policies, undermine public trust in government and evidence, and promote public alignment with their own messaging and preferred solutions. To counter such tactics, public health professionals need to gain a better understanding of the strategies unhealthy commodity industries utilize to deflect attention from their underlying health-harming intentions, especially through more novel platforms like social media.

## Background

### Introduction

The United States (US) remains the only high-income country (HIC) that does not provide universal health insurance coverage to its citizens [1,2]. Approximately 31.6 million Americans are uninsured and 40 million do not have adequate insurance coverage [3,4]. Compared with other HICs, the US health system ranks last overall in access to care, administrative efficiency, equity, and health outcomes [1] despite spending the most overall and per capita [5,6]. The US spent nearly 17% of its gross domestic product (GDP) on health care expenditures in 2019, which is almost twice as much of its GDP as other HICs [1].

Health care spending has been rapidly increasing in the US since the mid-1980s [7], and yet, this investment has not resulted in congruent improvements in health outcomes for Americans. Despite spending the most money on health care than any other country, the US has some of the worst health outcomes [4,7]. Americans have the lowest life expectancy of their peers in HICs, and experience the highest chronic disease burden, suicide rates, number of hospitalizations from preventable causes, and rate of avoidable deaths [4]. Racial and income-related disparities exacerbate these outcomes even further. Life expectancy among non-Hispanic black Americans (74.8 years) is 4 years lower than for non-Hispanic whites (78.8) [4,8], and between the poorest 1% and richest 1% there is a life expectancy difference of 14.6 years, which is expected to worsen as income inequality gaps widen [7,9].

### Private health care: Driving up health care costs in the United States

The US health care system underperforms against peer countries, generates large health disparities, and is also by some margin the most expensive. Health care dollars are primarily spent on hospital care (33%), followed by professional services (26%), long term care (13%), and prescription drugs (9%) [10]. Insurance covers these expenses only in varying degrees. Out-of-pocket spending then becomes necessary for Americans seeking care, with 38% of out-of-pocket expenditures going towards professional services and 13% towards prescription drugs [10]. As of 2020 private health insurance provided 66.5% of coverage in the US, compared with 34.8% public coverage [11]. The most common subtype of health insurance coverage was employment-based insurance (54.4%), followed by Medicare (18.4%), Medicaid (17.8%), direct-purchase coverage (10.5%), TRICARE (2.8%), and Department of Veterans Affairs coverage (0.9%).

Without a single-payer system and more stringent regulation of private health corporations, health care costs can vary widely across the US. Medicare spending differs from private coverage in that prices are set administratively instead of through decentralized negotiations between providers and payers [10]. Differences in health care spending within government-negotiated Medicare varies by utilization of the health system, whereas spending per privately insured person can be three times higher in parts of the country than others (even when controlling for disproportionate representation of elderly people using the health system more) [10].

Overall, a lack of universal health care (UHC) in the US was identified as the foremost reason for having the poorest health system performance among HIC in a 2021 Commonwealth Fund report [1]. The US ranked last in access to care, which included measurements of health care affordability and timeliness. Large disparities were also observed between income groups for all measured dimensions of access to care, particularly larger financial barriers to accessing medical and dental care, medical bill burdens, difficulty obtaining after-hours care, and use of virtual care to facilitate patient engagement [1]. The Commonwealth report’s foremost recommendation to improve access to care in the US was expanding and strengthening insurance coverage, which involves switching to more universal coverage policies and capping out-of-pocket medical costs.

### Conflicts of interest: Profits and health

Within this profit-driven health care system, private health care companies have a lot to lose with the implementation of UHC in the US. The for-profit private health insurance market generates approximately $670 billion annually [12]. Without government regulation, insurance companies and other essential elements of healthcare, such as pharmaceuticals, hospitals, private equity, and other financially motivated actors, can unilaterally set prices on care provision, hospital services, and prescription medication—the very elements contributing to the largest proportion of health care spending in the US. It is also not within their financial interest to expand coverage for everyone, which would include taking on 31.6 million new insurance-subscribers who may not be able to afford premiums or have poor health and higher coverage costs [13]. Despite estimates that implementation of UHC would reduce national health care spending by 13.1% annually [2], like other large industries, for-profit health care providers are resisting interventions that would constrain their profits. However, the nature and extent of their activities to do so are not yet well described.

### The Partnership for America’s Health Care Future: Lobbying to obstruct universal health care

In the leadup to the 2020 presidential election, policy options like Medicare for All, Medicare buy-in, and the public option all became hotly debated topics among Democratic Party presidential nomination candidates. While originally lauded only as aspirational at best, Senator Bernie Sander’s Medicare for All bill began to gain traction, with prominent Democratic candidates endorsing the bill, including Senators Kamala Harris of California, Cory Booker of New Jersey, Kirsten Gillibrand of New York, and Elizabeth Warren of Massachusetts [14]. Many of the same senators endorsed more modest UHC proposals as well, such as Medicare buy-in and Medicaid buy-in. In response, a coalition was quickly assembled, initiated by Chip Kahn, President and CEO of the Federation of American Hospitals (one of the most powerful hospital lobbies in the US), in an effort to stop UHC proposals progressing from campaign platforms to items on legislative agendas [12].

The Partnership for America’s Health Care Future (PAHCF) is a multi-million-dollar coalition that was formed in 2018 by the hospital federation and two powerful lobby groups, America’s Health Insurance Plans and the Pharmaceutical Research and Manufacturers of America [14,15]. The coalition consists of 124 members across the country, including profitable and powerful members like the American Medical Association, American Hospital Association, and Blue Cross and Blue Shield [16]. The name of the coalition has been described as intentionally nondescript [12], so as to not reveal its membership.

In response to debate around Medicare for All and related policy matters, members of PAHCF collectively spent $143 million USD on lobbying in 2018 alone [17]. In the spring of 2019, PAHCF announced that it was launching a “substantial, six-figure digital advertising campaign to inform the American public about ways to protect and strengthen [America’s] existing health care system” [18]. The announcement claimed that the campaign would be designed to warn Americans “that a one-size-fits-all health care program – whether called Medicare for All, Medicare buy-in, single payer or public option, will lead to higher taxes and less patient choice for every American family” [18]. Announcements of this type suggest such campaigns exist primarily to oppose expansions of single-payer health care system policies.

### Private health care as a commercial determinant of health

It appears that private health care companies’ business and political activities are consistent with the characteristics of the commercial determinants of health (CDOH). The CDOH are defined by Gilmore et al. as “the systems, practices, and pathways through which commercial actors drive health and equity” [19]. Impacts of CDOH are societally felt from micro to macro levels: from the individual consumer, their health behaviours and choices, extending to global levels of consumption, and the politics and economics of increasing globalisation. Within the existing health-services consumption landscape in the US, PAHCF is exercising their influence through all four characteristic corporate channels: marketing, enhancing the acceptability and desirability of private health care (an unhealthy commodity); lobbying, attempting to create UHC policy barriers; engaging in corporate responsibility strategies by forming a coalition to protect their individual corporate reputations and deflecting attention away from the coalition’s true mission; and utilizing their extensive network throughout the country to amplify the coalition’s influence [20]. Synergistically, these channels work to boost corporate reach, and amplify outcomes, negatively affecting consumers, their health and environment.

These strategies undertaken by unhealthy commodity industries (UCIs), such as tobacco, alcohol, and ultra-processed food, mirror the activities being undertaken by the private health care industry. This includes political tactics, shaping the preference of the public [21], and misrepresenting legislative intent of public health policies [22]. Most CDOH research to date focuses on the practices of UCIs, but recent analyses seek to expand this to include other industries, such as gambling, motor vehicles, and pharmaceuticals [23]. This study seeks to demonstrate how private health care industry lobbying is an example of wider CDOH activity, through the example of PAHCF campaigns on Meta platforms.

### Partnership for America’s Health Care Future’s Meta advertisement campaign

Social media platforms offer powerful targeting tools to advertisers so that their political or business campaigns can reach defined demographics [24]. Meta allows targeting of specific location (cities, communities), demographics (age, gender, education, job title), interests, consumer behaviour, and connections between users [25]. By running their advertisements through Meta and its popular social media platforms (Facebook and Instagram), PAHCF are able to specifically target large and highly specific audiences with their campaign.

Political advertising is a particularly important form of media messaging for agenda-setting and influencing public attitudes [26]. Combined with traditional media methods, political advertising delivered through social media channels may have a synergistic and amplifying effect on shaping public discourse (and inevitably legislative agendas) through increased frequency of exposure and targeting of messages [27], travel and spread of content across social networks, and creation of echo chambers [28]—though this has not yet been thoroughly explored. This is particularly concerning considering that Meta continues to allow false statements in political advertisements and refuses to participate in overseeing the truthfulness of content displayed on their sites [29], perpetuating and amplifying the spread of mis- and disinformation online.

In their announcement for their digital advertisement campaign launch, PAHCF state that “digital ads are [the] next step in educating Americans on ways to strengthen [the] health care system” [18]. Better understanding of how their tailored messaging might have the potential to distort public views of UHC—and more generally how it can inform perceptions of health, health risks and behaviours—requires a thorough analysis of PAHCF’s advertisement campaign contents and themes.

### Study purpose and overview

The aim of this study is to investigate the content and themes utilized by PAHCF in their Meta ad campaign, and strategies used to appeal to users to vote against the implementation of UHC policies in the US. This study sought to achieve several objectives. Firstly, the development of a qualitative coding framework to ascertain how information is presented by PAHCF to convey their intended meaning and messages, and to identify overarching categories and themes within the Meta advertisement campaign. From these, this study aimed to determine the strategies used by the campaign to appeal to certain demographics. Further, analysis of the coded data seeks to gain a better understanding of content, themes, and strategies utilized by PAHCF (and other commercial-actors) to advance their policy goals. This analysis helps us to understand how tailored messaging and campaigns might impact on public views of UHC policies, and more general perceptions of health, health risks and behaviours. It contributes to research on mis- and disinformation spread, and how it can influence health behaviours. Finally, this study seeks to contribute to the body of evidence that demonstrates the need for better understanding of strategies used by health harming actors and industries, the need for transparency and accountability of sources, social media as a vehicle for CDOH, and how to create effective counter-messaging [30] that informs the public and inoculates them against mis- and disinformation spread.

## Methods

### Ethics statement

The London School of Hygiene and Tropical Medicine Research Governance & Integrity Office determined that ethical approval was not required for this study (ref 27010).

### Data collection

Paid advertisements by the PAHCF Facebook page were identified on the Meta Ad Library, resulting in the retrieval of 1675 paid advertisements and its associated metadata [31]. The Meta Ads Library is an open directory of advertisements running on Meta platforms. The Library archives political or social cause advertisements and provides basic ad information (date ran, advertiser, low and high spend estimates, advertisement media, and text) and engagement metrics such as low and high viewer impressions, which measure how often your ads were on screen for your target audience [32]. The collected advertisements were shown to Meta platform users from 7 May 2018–12 September 2021. The information provided is supplied voluntarily and at the discretion of Meta; data scraping was required to collect this information, and researchers are limited by the information companies are willing to provide. Therefore, other data or engagement metrics may exist which are not accessible or disclosed. For example, we cannot report or examine if or how ads are targeted by detailed demographic or interest categories.

The media content was manually collected by screen recording (videos) and screenshots (images). This methodology is similar to the techniques used by Jamison et al. [33] and Zenone and Kenworthy [34], and also benefits from having a larger sample size of advertisements. Advertisements that used the same image or video with paired text, and were released at the same time, they were placed together in a subgroup. For example, if an advertisement was comprised of the same image and text and displayed to Meta users within the same time period, they were considered representative of the same advertisement content and put in the same subgroup. In total, the complete library of 1675 advertisements was organized into 486 unique subgroups.

### Analysis

To ascertain the themes and patterns within the visual and textual media of PAHCF’s advertisements, a content analysis was determined to be the most suitable methodology. Content analysis enables qualitative researchers to make systematic, replicable and valid inferences from text or other media (including images, videos, sounds, and symbols) to the contexts of their use [35]. It has previously been applied to analyse advertising themes as well as political campaign messaging [36], making it an ideal methodology to apply to PAHCF’s advertising campaign.

Advertisement data was uploaded to Dedoose [37], a qualitative and mixed methods research software, for analysis. Data from the representative 486 advertisement subgroups were uploaded to facilitate coding. Each advertisement media file (image or video) was assigned a file number that would be matched with the original file description in the main project file.

Advertisement media were primarily reviewed by the first author to develop a qualitative coding framework to determine content themes and categories. An inductive approach was used to develop the qualitative coding framework. This approach was chosen to enable research findings to be evaluated and determined from the frequent or significantly observed themes in the advertisement data [38]. This methodology also supports the aims and objectives of this study, in that inductive approaches enable the condensing of complex and varied data (such as images and videos) into a brief, summary format which can be used to establish links between research objectives and summary findings, and also contribute to the development of a theory about the underlying structure of themes and processes which are discernible in the data [38]. An inductive approach was also purposefully chosen to avoid key themes from being obscured due to preconceptions in the data analysis procedures sometimes imposed by deductive data analysis [38], particularly for less-explored research areas. Semantic and latent coding were purposefully utilized in analysis to identify both the descriptive and interpretive aspects of the data, respectively, to capture both the explicit and underlying content and themes that formed the advertisement campaign [39].

Initial content and themes identified were developed through the first round of open-coding. The coding framework was then refined through discussion with fellow researchers (supervisor and subject-matter experts), as well as by referencing existing literature and analytic notes taken while reviewing advertisements. The first author coded each advertisement, and any uncertainty about media content was resolved through discussions and recoding, as necessary. Exported coding data were subsequently matched with other advertisements metadata within the same subgroup in the main project spreadsheet.

An iterative process was used to develop categories and analyze the content and themes identified in PAHCF’s campaign. Categories were determined by the first author after discussions with co-authors. This process determined three major categories for content and thematic analysis: policy targets, claims and themes, and appeal groups.

## Results

### Campaign spend, impressions, and demographics

The PAHCF campaign received between 32,596,000 and 40,706,329 impressions and spent between $842,700 and $1,168,928 USD on advertisements.

### Advertisement composition

Each advertisement followed the typical posting format available on Meta platforms. At the top of each advertisement, the “Partnership for America’s Healthcare Future” is bolded in black text to the right of its brand/profile picture, which is a circle that encloses an illustration of two hands shaking (one hand is a light blue and the other a coral-red colour; these colours are likely in reference to the two political parties in the US, Democrats and Republicans, respectively). Indication that content is an advertisement is conveyed to Meta users by smaller, greyed-out text that reads “Sponsored - Paid for by the Partnership for America’s Healthcare Future”, below the larger title text and image. Campaign text is featured directly below this, where campaign messages are conveyed. Visual media, such as images and video, comprise the lower half of each advertisement and depict representative imagery related to the caption above. The image component of the advertisements contained a wide-range of visual media content, including images of people, medical, or government imagery with text overlaid; videos of people relaying scripted messages; and/or illustrations or infographics to convey PAHCF’s chosen messages. Some advertisements also feature links to PAHCF’s Meta profile page or their external website, and invite users to “Learn more”. It is important to note that advertisement and user posts are comprised of very similar elements, and the type of post (e.g., advertisement or user) may not immediately be distinguishable to a viewer, helping advertisements blend in seamlessly among user-generated content. PAHCF advertisements can be viewed on their Meta Ad Library page [31].

### Policy targets

Five major policy targets were referenced in PAHCF’s advertisement campaign, non-exclusively. A “public option” was featured in 56% (n = 943) of advertisements, Medicare for All in 44% (n = 732); new government insurance systems in 34% (n = 565), Medicare buy-in in 29% (n = 488), and Medicare at 60 in 1% (n = 21). Many advertisements falsely equivocated the public option, Medicare for All, and Medicare Buy-in as the same policy options. Samples of this include advertisement text that reads “Medicare for all, Medicare buy-in and a public option will all mean the same thing for American patients”, or “Medicare for All = Medicare Buy-In = The Public Option”. Multiple policy options were frequently included in one advertisement, and are sometimes representatively lumped together by describing them as “Medicare for all bills” that would “increase taxes on hardworking families—potentially doubling Americans’ income taxes”. Personal testimonies, often paired with images or videos of the person saying the campaign messaging (such as how a “new government insurance system” would “force” Americans to “pay more to wait longer for worse care”) is another featured format. See Table 1 for summary of excerpts, impressions and spend for policy targets within the campaign.

**Table 1:** Policy Targets – Excerpts, Impressions, and Spend.

*Policy Target* Names of proposed universal health care policies identified throughout the advertisement campaign.			
Policy Target	Excerpt	# Impressions	Total Spend ($USD)
Public option (*n* = 943; 56%)	*Medicare for All*, *Medicare buy-in*, and the *public option* won’t lower our health care costs.	17,362,000– 21,167,057	$395,000– 554,557
Medicare for All *(n = *732; 44%)	Whatever they call it, *Medicare for All*, *buy-in*, or a *public option*, it would mean the same thing: a one-size-fits-all government takeover of the health care system.	14,880,000– 19,105,269	$453,300– 618,968
New government insurance systems *(n = *565; 34%)	Under a *new government insurance system* like the *public option*, patients like Meggie will be forced to pay higher taxes, premiums, all for lower-quality care.	10,028,000– 12,242,435	$310,800– 433,535
Medicare buy-in *(n = *488; 29%)	Next time you hear *Medicare for All*, *Medicare buy-in* or a *public option*, know that all of these proposals put Americans’ health care at risk.	6,989,000– 8,630,512	$129,600– 196,012
Medicare at 60 *(n = *21; 1%)	‘*Medicare at 60* could mean higher costs, lower quality care, and less access to care. Let’s protect the Medicare coverage and care our seniors rely on-not put it at risk.’	1,879,000– 2,213,979	$33,200– 40,679

### Advertisement claims and themes

The analysis identified twelve major ad claims and themes that cut through these policy targets. These were organized into five categories, based on similarity or analogousness (as determined through discussions between the first and second authors). The five overarching categories of ad claims and themes were determined to be: 1) the negative impacts of UHC policies on health care in the US; 2) infringement on individual choice and rule by the state; 3) misrepresentation of legislative intent; 4) promoting partnerships and fixing the current system; and 5) appealing to audience interests. How each of the respective twelve major ad claims and themes were represented, their frequency of appearance in the advertisement campaign, and which of the five categories they belong to is described below. Multiple themes across categories could be presented within one advertisement. The twelve identified claims and themes were depicted throughout the campaign using images or videos paired with captions often showing concerned citizens represented by a mixture of men and women; women were often placed in domestic settings (such as at home in their kitchen or with their children), while men were often depicted at their place of work. Captions and images or videos of testimonials from patients or persons of authority (such as a nurse or city counsellor) were also employed. A summary of excerpts, impressions, and spend for each identified claim and theme are provided in Table 2.

**Table 2:** Advertisement Claims and Themes – Excerpts, Impressions, and Spend.

Claims & Themes	Excerpt	# Impressions	Total Spend ($USD)
*Negative impacts on health care in the United States* Claims that universal health care policies will increase difficulties for Americans to access and afford health care.			
More expensive, higher cost *(n = *988; 59%)	‘These government-controlled proposals could *double everyone’s taxes*.’ ‘With government run insurance, *we’d pay more* to wait longer for worse care.’	17,307,000– 22,232,013	$496,700– 682,912
Threaten access to quality care *(n = *812; 48%)	‘Paying higher taxes or higher premiums *for worse care*? It’s time to say no to proposals we can’t afford.’ ‘It’s very important *that I always have access to the doctors* that I need so that I can have a good quality of life.’ ‘Medicare for all and government health care will result in higher taxes and *lower quality care*.’ ‘It is very concerning to me that Medicare for all and some of these other government run insurance systems *could cause hospitals and NICUs to close across the country*, and that is very dangerous.’	15,139,000– 19,557,189	$403,400– 562,888
Increased wait times *(n = *430; 26%)	‘A public option and government run insurance systems will mean *longer wait times*, worse care, and higher taxes. It’s wrong.’ ‘These new bills will create *longer wait times* for less care.’	7,014,000– 8,734,570	$183,200– 257,170
*Infringement on Individual Choice and Rule by the State* Imposition of the state on individual choices, and threats of the government taking away health care autonomy.			
One-size-fits-all care (*n = *745; 45%)	‘My fear about the public option is that it is going to be a *one-size-fits-all* approach. They could dictate what doctors we see, what hospitals we go to.’ ‘A *one-size-fits-all health care system* will mean all Americans have less choice and control over their doctors, treatments, and coverage.’ ‘[These new bills] are a *one-size-fits-all* solution for all Americans: old or young; sick or healthy.’	14,638,000– 17,893,255	$398,500– 545,455
Limited choices, less control (*n = *612; 37%)	‘Under these bills *government officials would control your health care coverage* from the doctor you see, to the benefits covered, even your reproductive health.’ ‘Medicare buy-in will *take away choice and control* for millions of Americans.’ ‘The concern about public option is my fear with anything the government runs: *they take total control of it*. I’m not sure they should get into the business of deciding what kind of health care I should have.’	9,956,000– 12,151,388	$225,100– 320,488
Warnings of big government and bureaucracy (*n = *409; 24%)	‘My biggest fear for government run health care or Medicare for All, your child becomes a statistic and not an individual. We *don’t need the bureaucracy of government-run health care* making the decisions.’ ‘Medicare for All and other government run insurance systems puts *politicians and bureaucrats in control of our health care*.’ ‘I don’t want Washington *politicians and bureaucrats messing with our benefits*.’ ‘I want my doctors to make decisions for me, *not the government*.’ ‘Unaccountable to voters, these *bureaucrats will have unchecked powers* to make decisions that impact you.’	11,911,000– 14,325,591	$278,200– 368,691
*Misrepresentation of Legislative Intent* Suggestive that proposed universal health care policies will be detrimental to public health, the economy, and society.			
The ‘real truth’ about policy (*n = *415; 25%)	‘The politicians may call it Medicare for All, Medicare buy-in or the public option, *but it means the same thing*: we pay more, to wait longer for worse care.’ ‘It might be hard to believe, *read it yourself*: these bills would take power out of the hands of patients and doctors and put into the hands of the government, eliminating the current health care system, Medicare and Medicaid in the process.’ ‘And these bills don’t even include a way to pay for the trillions of dollars in costs—probably because *they don’t want voters to know* it will mean higher taxes.’	10,400,000– 12,533,585	$271,100– 360,885
*Promoting Partnerships and Fixing the Current System* Direct suggestions for private health care and public programs work together, and to make the current system work.			
Private health care and public programs working together (*n = *232; 14%)	‘Let the *free market and public programs work together* to lower costs, increase patient choice, and improve quality—that’s how we’ll achieve the health care future Americans deserve.’ ‘How do we fix health care? It’s going to take the full efforts of the *free market and public programs working together*.’ ‘*Working together* to Improve Quality health care – The Partnership for America’s Health Care future.’	5,709,000– 6,866,768	$141,300– 188,468
Fix what is broken, but keep current system (*n = *207; 12%)	‘We need to *fix what’s broken, not start over*.’ ‘That’s why *fixing Medicare before expanding* it makes sense.’ ‘It’s time to *build on what’s working and fix what’s broken*, not start over.’	4,257,000– 5,198,793	$69,300– 100,293
*Appealing to Audience Interests* Messages designed to appeal to and promote alignment across diverse population groups and their interests			
Americans deserve quality health care (*n = *328; 20%)	‘What Americans *deserve* is health care that gives patients more choice, more control, and better quality care at a price they can afford.’ ‘Americans *deserve* more options, not just one.’ ‘American families *deserve better* than a new government health insurance system that will force them to pay more, to wait longer, for worse care.’	7,232,000– 8,785,672	$139,000– 196,172
What everyone wants (*n = *170; 10%)	‘We come from different walks of life but *we agree on one important thing*: we don’t want to be forced into a one-size-fits-all government insurance system.’ ‘*Americans want* … private coverage to work together with Medicare and Medicaid.’ ‘*Every American wants* health care they can afford and trust. The public option does the opposite.’	4,478,000– 5,396,830	$96,400– 131,930
Temporal threats/COVID-19 (*n = *26; 2%)	‘#Working together: PhRMA members are developing new therapies and treatments for those infected with *COVID-19*.’ ‘America’s health care partners are united by a common goal to stop the spread of *COVID-19*—#WorkingTogether to help Americans get healthy and stay healthy.’	1,331,000– 1,584,975	$22,400– 28,875

(1) The negative impacts of UHC policies on health care in the US

This was the most frequented category of claims and themes, and included claims that UHC policies would: impose more expensive and higher costs (59%, n = 988), threaten access to quality care (48%, n = 812), and increase wait times (26%, n = 430). Often all three messages would be combined in one advertisement, claiming that introduction of UHC policies would force Americans to “pay more to wait longer for worse care”. Advertisements also often included threats of higher taxes and unexpected costs, such as captions claiming a possible requirement of “more than doubling Americans’ taxes”, paired with images of representative Americans looking through their bills with concern, or showing a parent with their children with indications of the existing high burden costs for families (such as large grocery bags, or being placed in medical or recreational activity settings). Claims were often made hypothetically, including captions like “Research shows that Medicare for all would cost $32 trillion and could require more than doubling Americans’ taxes”, without any citation or demonstration of evidence. Threats to access for quality care were conveyed through captions using fear-mongering language, such as how the proposed UHC policies “threatens access to care”, that Medicare for All “will result in…lower quality care”, or even a caption and video of a neonatal intensive care unit nurse claiming that “these other government run insurance systems could cause hospitals and NICUs to close across the country, and that is very dangerous.” Longer wait times were often described as an inevitability of implementing any of the UHC policy options.

(2) Infringement on individual choice and rule by the state

These themes described how UHC policies would impose one-size-fits-all care (45%, n = 745), limit choices and offer less control by citizens over their own health care (37%, n = 612), and contained warnings of big government and bureaucracy (24%, n = 409). These themes often utilized fear-mongering or threatening language to convey that Americans would lose their health care autonomy to bureaucrats if UHC policies were implemented, and that politicians were likely to impose their personal will on the public. Images and video often paired with this messaging include personal testimonials from citizens who “don’t want Washington politicians and bureaucrats messing with our benefits”, and animated infographics demonstrating how the bills will “take power out of the hands of patients and doctors [and] putting them into the government”. Ads within this theme also suggested irresponsible spending by politicians and worsening federal deficits that the public would have to pay for. Advertisements with these themes also implied that it was the individual’s responsibility to manage their own health care, and to ‘say no to the public option’.

(3) Misrepresentation of legislative intent

These advertisements directly claimed that PAHCF messages were offering citizens the ‘real truth’ behind UHC policies (25%, n = 415), in that they would be harmful to public health, the economy, and society. Related to the previous category of claims and themes, these advertisements promoted deeper mistrust of government (while posing themselves as a more trustworthy provider of information), with messages implying that UHC policies were deceptive and not in the public’s best interest. The misrepresentation of legislative intent theme often employed captions that diminished the trustworthiness of politicians, using text such as “[politicians] don’t want voters to know…”. These messages were frequently delivered through images or videos depicting a person of authority revealing these ‘real truths’ to their audience, such as a woman walking through a busy office in business attire describing how “the politicians may call it Medicare for All, Medicare buy in, or the public option, but it all means the same thing”; or a testimonial from a city counsellor “who knows that Americans deserve better”.

(4) Promoting partnerships and fixing the current system

Advertisements included direct suggestions that private health care and public programs worked together to make the current system work (14%, n = 232), and argued for fixing what was broken but keeping the current system (12%, n = 207). These messages were often presented with a positive, harmonious connotation, in opposition to the way advertisements portrayed the government. These themes also often used softer imagery or narration, like infographics or animation, compared with themes within other categories. Working together was offered as an acceptable, simple solution to complex health care problems.

(5) Appealing to audience interests

Finally, appealing to audience interests included suggestions that Americans deserve quality health care which public options cannot provide (20%, n = 328), that voting against Medicare for All is what everyone wants (10%; n = 170), and sympathizing with shared public concerns for being protected against the spread of COVID-19 (2%; n = 26). These messages aimed to promote alignment across diverse stakeholder groups. Captions within these themes often used phrases like “Americans want” or “Americans deserve better”, and were paired with images that were representative of a diverse set of stakeholders, including “American families” or “hard-working Americans” in their place of work.

### Targeting specific appeal groups and demographics

The campaign content reflects that it was designed to appeal to a wide range of population groups. Groups were often representative of ‘everyday’ Americans, who represent large demographics within their advertising audience. Advertisements regularly featured threats that single-payer health options posed to specific groups, always encouraging them to vote against these public health policies. Appeal groups included: American families (n = 495; 30%), Americans (n = 466; 28%), moms (n = 258; 15%), patients (n = 124; 7%), seniors (n = 62; 4%), rural communities (n = 46; 3%), and Hispanic/Spanish-speaking Americans (n = 10; 2%). All appeal groups were identified directly from advertisement media or text. Imagery or video used for these advertisements showed individuals who were visually representative of each of the identified stakeholder groups (such as moms with their children, senior citizens, or a person speaking Spanish), and/or placed within their respective representative contexts (such as in medical facilities, or in a rural community with a tractor in the background). Messages targeted specifically at ‘Americans’ invoked a sense of national patriotism, while other groups included use of personalized narratives and storytelling often employed to representatively emphasize how adoption of a new government insurance system would increase difficulties faced by groups who are deserving, hard-working, and already struggling with illness or accessing health care. A summary of excerpts, impressions, and spend of appeal groups is provided in Table 3.

**Table 3:** Appeal Groups – Excerpts, Impressions, and Spend.

*Appeal Groups* Groups directly identified in from advertisement media or text, paired with messages invoking a sense of patriotism or suggestions they would be negatively affected by the implementation of universal health care policies.			
Appeal Group	Excerpt	# Impressions	Total Spend ($USD)
American families (*n* = 495; 30%)	‘Too many patients still can’t get the care they need, and *for too many families* costs have been a financial anchor.’ ‘It will also mean trillions in higher taxes for *hard-working families*.’ ‘*American families can’t afford* the consequences of the public option.’	12,277,000– 15,827,506	$448,200– 593,705
Americans *(n = *466; 28%)	‘*Americans* deserve more options, not just one.’ ‘The public option is a one-size-fits-all government health insurance system that would mean higher costs and fewer choices *for Americans*.’	10,068,000– 12,224,535	$223,700– 308,935
Moms (*n = *258; 15%)	‘*Like every mom*, I care a lot about my family’s health care. The new government-controlled health insurance systems politicians are pushing are a real threat to our health care coverage, and yours.’	5,626,000– 7,819,743	$281,800– 376,342
Patients *(n = *124; 7%)	‘We thought we had seen what a worst-case Medicare for All bill would look like, but the new bills in congress *are worse for patients than we thought possible*.’	1,861,000– 2,299,876	$29,500– 45,376
Seniors *(n = *62; 4%)	‘*Seniors* depend on Medicare and expect politicians to keep their commitment and protect our benefits. That’s why fixing Medicare before expanding it makes sense. When the politicians can’t pay for it, they’ll raise our taxes and cut [seniors’] Medicare.’	1,556,000– 1,850,938	$23,900– 33,738
Rural Communities *(n = *46; 3%)	‘The public option could *endanger rural hospitals*, hurting families and *entire communities*.’ ‘A new government insurance system poses a major threat to *rural communities*.’	1,890,000– 2,261,951	$55,600– 66,751
Hispanic/Spanish-speaking Americans *(n = *10; 0.6%)	‘*Planes de salud del gobierno significan más impuestos y peor cuidado.*’ <<Government health plans mean higher taxes and worse care.>>	50,000– 71,990	$1,000– 1,990

## Discussion

### Key study findings

Content analysis offers a critical way to deconstruct PAHCF’s advertisement campaign, and enables the identification of the categories, themes, and tactics they intentionally use to distort public perception of UHC in the US. Three major areas of focus emerged from the coding framework and analysis of campaign content: UHC policy targets, campaign claims and themes, and targeted appeal groups. Ubiquitous identification of the five policy targets throughout the campaign served to orient American audiences to focus on how implementation of any of the UHC policies could negatively impact them. Collectively, all campaign claims and themes are constructed to work together to mutually reinforce one another, representing that the proposed UHC policies will be detrimental to public health, the economy, and society [40]. In contrast, PAHCF’s own advertising and lobbying activities are framed as being in the best interests of the public. Direct identification and representation of seven different demographic groups is intended to appeal to and promote alignment across diverse population groups with PAHCF’s own messaging and preferred solutions. Being paired with messages on how potential implementation of UHC policies would negatively impact each of these groups, advertisement content has the potential to exploit existing vulnerabilities of targeted demographics, such as economic vulnerability or lack of access to quality care.

The contents, themes, and strategies prevalent throughout PAHCF’s advertisements are collectively being utilized to convince a large audience of Americans about the negative impacts inherent in UHC policies, and to advance their policy goal of pre-empting the implementation of any UHC policy in the US. These findings show that the private health care industry is participating in and adding to wider CDOH activities. These findings also build on previous CDOH research to recognize and include other industries as contributors to CDOH, through their use of discursive tactics and political strategies, not unlike UCIs, to protect their profits to the detriment of public health.

### Commercial determinants of health: Discursive strategies

Analysis of PAHCF’s campaign contents and themes shows how it utilizes tactics common among UCIs to operationalize their profit-protecting agenda. In particular, the application of Ulucanlar et al.’s Taxonomy of Discursive Strategies (TDS) demonstrates the purposeful intentions behind the strategies PAHCF uses in their campaign, and wider CDOH activity [40]. The TDS was constructed to examine tobacco industry political activity, particularly its interference in the implementation of evidence-based policies to reduce tobacco use [40]. While their taxonomy was developed to describe strategies employed by the tobacco industry, as they suggest, their discursive strategies are applicable to political activities by non-tobacco industries that also threaten the public’s health [40]. Like tobacco, the private health care industry appears to be posing a substantial impediment to progress towards policy implementation—in this case, UHC. The TDS proposes that industry’s overall discursive strategy is to exaggerate the potential costs of a proposed public health policy while simultaneously dismissing (or denying altogether) its potential benefits. These strategies collectively aim to build a comprehensive and credible narrative that UHC policies are undesirable, incorporating arguments tailored to be inclusive of diverse population groups and social domains. The misleading public information campaign seeks to sow fear, manufacture doubt, and increase uncertainty [41] towards an entire spectrum of UHC policy reform efforts. The PAHCF campaign also utilizes a key feature of the TDS in that its messages are designed to appeal to diverse sociopolitical domains and groups, and that their messages are articulated not only by PAHCF, but through representative third parties (including citizens) within their campaign. This constructs the appearance that opposition to proposed UHC policies is not in the profit-oriented self-interests of PAHCF, but rather something members of the public are genuinely concerned about. Three major discursive strategies make up the TDS: expanding and creating potential costs, containing and denying benefits, and absent benefits.

Expanding and creating potential costs involves exaggerating the costs of proposed policies. The claims and themes identified in PAHCF’s campaign are exemplary of this. Firstly, this strategy seeks to expand the type and reach of unanticipated costs to the economy and society, and creates new costs in each of these domains [40]. The negative impacts of UHC policies, including the threat of higher costs, is an illustrative example of economic costs (unanticipated and created); while other negative impacts (such as threatened access to quality care and increased wait times) and infringements on individual choice and rule by the state (including one-size-fits-all care and limitations on choice and control by citizens over their own health care) serve as examples for potential costs to society.

Secondly, expansion and creation of potential costs argues that unintended benefits will be provided to undeserving groups—in this case, bureaucrats gaining increased control over citizens, suggested irresponsible spending by politicians, and worsening federal deficits that the public will have to pay for. This also calls to mind the adoption of nanny-state rhetoric as a strategy, another common tactic used across UCIs [42], such as the Institute of Public Affairs’ “Ten worst nanny state policies” [43], and the sugar sweetened beverage industry’s dissemination of misleading information related to taxation effects [34,44] and claim that public health policies interfere with personal freedom [45]. The PAHCF ads seek to shape public perceptions around the role of the government in health care, implying that its involvement will raise taxes, and take away Americans’ control over their health. As these messages are delivered through the visual or textual media of each advertisement, PAHCF is able to dissociate itself from the notion of ‘big insurance’ and is able to instead align itself with ‘everyday’ American citizens. It also places itself in a positive connotation through their messages that suggest their willingness to partner with existing public programs, included in the themes of working together and making the current system work. Third, this strategy argues that the proposed policy will have unintended public health costs—particularly that Americans will have to “pay more to wait longer for worse care” under a UHC system, and that the policies overall are not in their best interests. This is also prevalent in themes that promote mistrust of the government and misrepresent the legislative intent of UHC polices. Their overall misrepresentation of legislative intent through use of logical fallacies, unsound arguments, and false equivocations also mirrors efforts by tobacco lobbyists in opposition to governmental regulation of their industry [22]. This also includes arguments that government regulation could lead to a ‘slippery slope’ of future government action [46], or that it could have ‘negative unintended consequences’ [47]. The use of the hypothetical exploits the psychological susceptibility to fear the unknown, encouraging the preference to stay within what is known [48]—or, within the current system. It serves to shift consumer attention towards the impacts of an imagined future, rather than on the flaws of the current system.

The second TDS strategy is the containing and denying of benefits, wherein industry seeks to dismiss or deny any potential benefits of the public health policy or their own costs, should implementation occur, using mutually reinforcing, interdependent arguments [40]. This is apparent in the themes identified to appeal to audience interests, such as Americans deserving quality health care, as well as direct identification and representation of seven appeal groups. Targeting of appeal groups in particular strategically expands PAHCF’s narrative focus towards the adverse impacts that would be experienced by more vulnerable members of society, such as patients, seniors, and members of rural communities—and entirely away from the adverse impacts that the private health industry would experience with the implementation of UHC policies. This is also where PAHCF engages in a key feature of the TDS, by using representative persons and citizens in their visual media (video or images) as conduits to articulate their campaign messages, giving the appearance that members of the public are the ones in opposition to proposed UHC policies, without any acknowledgement of the costs PAHCF would face if implemented. This is akin to tobacco activities that emphasize the negative impacts on deserving groups such as retailers and farmers, and deemphasizes private industry loss of profits. Instead, industry suggests that implementation of public health policies are posed as a public loss to the economy and society as a whole.

Finally, the third TDS is absent benefits [40]: this strategy wholly excludes from industry narratives the wider benefits that implementation of public health policies would commonly provide to the economy and society. This is discernible in that evidence that supports the benefits of UHC implementation in the US is entirely absent from PAHCF’s campaign content, claims and themes. Like other UCI campaigns, PAHCF frames their lobbying narratives to encourage opposition to public health policies to be in the general public’s best interest [34]. But perhaps what is most striking about PAHCF’s campaign is how its messages are in direct contradiction with existing evidence. Lack of UHC in the US was identified as the foremost reason for having the poorest health system performance among HICs [1]. The US spends the highest proportion of their GDP than any other wealthy nation on health care, and markedly produces the worst health outcomes—which are even worse still when considering racial and income-related disparities [1]. The majority of PAHCF campaign messages uphold that implementing UHC policies will lead to less affordable and less accessible quality care, and cause increased wait times. This flatly disregards that under the current system, the US currently ranks last in all three of these areas, compared to their HIC peers [1]. While estimated costs to implement UHC may seem high, requiring $3.034 trillion annually, this is actually $458 billion less than current national health care expenditures [2]. This can be further broken down into $210 billion savings on hospital care, $111 billion on clinical services, $224 billion on administrative costs and overhead, and $180 billion on pharmaceuticals [2]—translating to a 13.1% reduction in per-capita annual expenditure. Single-payer UHC instead has the potential to improve accessibility and cost-effectiveness of health care in the US [49]. The PAHCF campaign messages rely on the long-held perception that UHC is politically and economically impractical for the US, but this is simply not supported by evidence [2].

Furthermore, PAHCF’s campaign narratives are not only constructed to be absent of data about UHC policy benefits, but emphasize claims that UHC policies would be actively harmful to US citizens and broad interest groups who “deserve” better care. These narratives obfuscate the millions of citizens who are currently un-or under-insured, and harmed by their inadequate access to health coverage. It is estimated that uninsured Americans access health care at 50.1% of the rate of insured individuals [50], while underinsured utilize health care at 86% of the rate of insured individuals [51]. Studies have indicated that these patients may be more likely to have undiagnosed comorbidities and conditions compared with insured patients [52,53]. The lifesaving impacts of UHC policies in providing insurance to currently uninsured Americans is estimated to save 68,531 American lives, or (by averting premature deaths, particularly among adults aged 25–35 who are disproportionally represented as uninsured individuals) 1.73 million life-years annually [2]. More pertinent than any economic estimates, implementation of UHC in the US would save lives. However, this evidence is purposefully and entirely absent from PAHCF’s campaign, as it is in direct contradiction with the narrative they wish to convey.

One possible reason for this obfuscation is that the PAHCF campaign is engaging in a discursive strategy to curtail and limit those who are seen as truly deserving of high-quality health coverage. This strategy leverages a long-standing history in the US of selective deservingness when it comes to social and health programs, in which marginalized people and communities of colour have been strategically and relentlessly categorized as undeserving of help, while those who are white, more affluent, and in other privileged groups are portrayed as the ‘truly deserving’ [54]. Selective deservingness justifies exclusion from the body politic, as well as curtailed and limited social programs such as public health coverage [55]. By engaging in such strategies, PAHCF successfully defines a limited interest group (well-off, already insured) for health care policy-making in the US, while denying the deservingness of the un- and under-insured and the harms they have experienced.

Like the tobacco industry, PAHCF have constructed in their campaign an overarching narrative of a dysfunctional future that will result if the proposed policies are implemented, and widely disseminating this narrative to enhance its persuasiveness [40]—in this case, through nearly 41 million impressions of Facebook and Instagram ads. The TDS is a component of a larger framework which Ulucanlar et al. term The Policy Dystopia model [40]. While the TDS and Policy Dystopia model were originally constructed to examine tobacco industry CDOH activity, this study demonstrates that private health care, as a member of wider health-harming industries, are also using key strategies of the dystopia model to oppose public health policies [40]. This model also suggests that political tactics should be accounted for in analysis of wider CDOH activities, of which PAHCF has been identified as engaging in.

### Commercial determinants of health: Political tactics and preference shaping

Further consideration of the tactics that PAHCF engages in can also be approached using Madureira Lima and Galea’s Corporate Practices and Health Framework (CPHF) [21]. Their framework uses Steven Lukes’ three-dimensional view of power to systematically map practices deployed by commercial actors to promote consumption of their respectively produced commodities that cause disease and injury. The CPHF postulates that power, in its three dimensions, can be exerted through five vehicles, including the political environment and preference shaping—of which PAHCF’s tactics are exemplary.

Political tactics are employed to consolidate industry influence in political and public spaces. By targeting five UHC policies in their campaign, it attempts to control agenda-setting, decision-making, and even non-decision related to each of these policies by keeping statutory regulation at bay [17]. The PAHCF is attempting to protect their profits while avoiding regulation by advocating for the self-regulation of their industry, and offering to form voluntary partnerships between private industry and public programs, like the processed food industry [56]. It is also acting to delay implementation of potential regulations, like the sugar-sweetened beverage industry [34]. Lobbying is another political tactic identified by CPHF. It enables corporations to have access to policy makers unavailable to voters and public interest organizations [21], and thus increased ability to sway legislative agendas in their favour. Spending between $842,700 and $1,168,928 USD on this Meta campaign is in addition to the $143 million spent by PAHCF members on lobbying efforts in 2018 alone [17]; members include pharmaceutical companies who regularly participate in this type of corporate-influence behaviour [21], and is akin to other UCI behaviour, such as food and alcohol [42,57]. This is also indicative of an existing power asymmetry, as PAHCF members have far more corporate resources available to invest in lobbying and campaign efforts compared with public actors who might provide counter-messaging to the public to help shape perceptions around health behaviours [21]. This is also within a political environment where health care companies in the US spent $692 million on lobbying efforts in 2021 alone, and over half a billion each year for the last seven years [58].

The second dimension of CPHF that PAHCF engages in is attempting to shape public perceptions around the role that governments should play in health care in the US [21]. This is achieved by ensuring that any tension between the interests of the public’s health and their own are never perceived as being in conflict or constructed as social or political issues [21]. By offering placating, oversimplified solutions such as “working together” and maintaining the current system while committing to improving it, PAHCF is never required to confront that they are upholding the worst-ranked health care system among HICs to their audience. Portraying that private health care can provide ‘what everyone wants’ creates a false sense of social consensus around an unhealthful commodity, erroneously shaping public preferences, and avoiding public conflict [21]. Strategic coalition formation also achieves this by enabling individual corporations and organizations to hide behind an intentionally vaguely named front group [42], like The Center for Consumer Freedom [59] or The International Life Sciences Institute [60], so as to not reveal their membership and avoid becoming involved in open confrontations that could harm their corporate images or profits. PAHCF also employs public relations companies to help manage their image, and anticipate and avoid conflict [17]. As demonstrated through the CPHF, PAHCF is exerting its corporate power by using political and preference shaping tactics to perpetuate the current health system for their own commercial benefit. By constructing the narrative that all Americans will be negatively impacted by the implementation of UHC policies, PAHCF exploits the sentiments of grievance politics that are particularly prevalent in the US [61] to unite their audience in opposition to UHC policies. Overall, these tactics are utilized to shift public perceptions around the role of government as a regulator, as well as advocating for the role of personal responsibility [21]. Like a UCI, PAHCF is acting to redefine to the public which institutions are ‘threats’ (government, taxes, bureaucrats), which are ‘emancipators’ (PAHCF, private health providers, opposing UHC), and encourage stakeholders to unite across diverse appeal groups—against their own best interests [34].

### Amplification of the commercial determinants of health through social media

As we can see, the UCI strategies and tactics PAHCF are recycling in their campaign are not new, however, the platforms they are using to disseminate their messages are. Targeted social media advertising campaigns have the capacity to expand the number of consumers exposed to a product, and to shape the psychological and social predisposition to accept and endorse its consumption [21]. What is perhaps the most alarming feature about using social media in health-harming campaigns are the tools that enable advertisers to target users with their desired audience characteristics. Lobbyists from UCIs can determine which demographics they would like to see their political ads, which includes characteristics such as location, age, gender, interests, and consumer behaviour [25]. Campaigns are designed and deployed to specifically include those who are more likely to ‘like’ and share campaign ads, and potentially groups who are more vulnerable to their messaging. This is likely why PAHCF’s campaign was designed to focus on the economic vulnerabilities of American families (particularly low- and middle-class families), patients who are reliant on the accessibility of health services, seniors reliant on the Medicare benefits, rural communities that already suffer from reduced access to health care, and Hispanic-Americans who are have the highest rates of uninsurance of any race (and would be threatened by even further reduced access to care) [62]. This methodology is becoming more pervasive, as other health-harming industries also utilize social media and targeted marketing to promote their products and shape public and political discourse [34]. These methods are more powerful than traditional advertising practices and are outpacing the regulations that govern them [24]. Considering that PAHCF campaign advertisements received nearly 41 million impressions in total, and the false claims and themes each contain, this is indicative of mis- and disinformation spread about a health-harming industry on a mass scale.

This occurs within a wider context, in which Meta continues to refuse to regulate and oversee truthfulness in political advertising [29], further enabling of the spread of misinformation, forms of hateful speech, rhetoric, and polarizing content with minimal moderation [63]. Meta also allows advertisers to conceal their sponsors [34]: frequently the PAHCF logo only appears at the end of videos, and rarely in images; if sponsorship was declared in ad media, it was in small, hard to read font. These practices are resulting in a lack of transparency for social media users to be able to identify who is sponsoring and promoting advertisements targeted at them. Meta also does not disclose exact spends and impressions for every advertisement, further restricting transparency to the public and researchers who are investigating social media interactivity. And while Meta has recently announced that it intends to share more details about political ad targeting with researchers through their ‘Facebook Open Research and Transparency Project’ [64], this may be interpreted as an act of self-regulatory corporate social responsibility as Meta is only allowing researchers they approve to access this additional information, and have blocked researchers’ Facebook accounts and site access in the past [65].

It has also been argued that social media itself is harmful to health [66], and that their own business and political practices are consistent with the characterizing features of CDOH [24]. Social media companies rely on advertisements for revenue—and Meta one of the leading advertising platforms in the world (second only to Google), earning $115 billion in advertising revenue in 2021 alone [67]—putting the best interests of public health in direct conflict with social media companies’ profits. The current, unregulated business model of social media is exacerbating the effects of mis- and disinformation spread, erosion of democratic values and processes, and overall negative impact on broader determinants of health [24]. Political advertising using social media platforms as its vehicle for their messaging is proving be capable of delivering synergistically negative impacts on public health.

### Conflicts of interest and pollution of public health discourse

Overall, PAHCF’s use of discursive strategies, political tactics, and social media for their campaign are all methods applied to conceal the fundamental conflict of interest between corporate interests and public health [40]. Although a certain level of conflict of interest may be unavoidable in private-public partnerships, the likelihood of conflict is enhanced by deceit and/or manipulation and exacerbated by a lack of transparency [68–70]. Analysis has shown how PAHCF engages in deceitful tactics that intend to manipulate public perception of UHC policies, and strategically engage in wider CDOH activities and social media platforms to conceal the true intent of their advertising campaign. Well-funded and strategically crafted efforts to shape public perception on health and public health policies, like this one, can have negative cumulative effects and is contributing to the ‘pollution’ of health discourse [30]. It is essential for public health to acknowledge and be able to pre-empt this industry pollution through effective counterarguments [40]. This includes increasing awareness of the pollution of health discourses, and the methodologies employed by health-harming actors—of which, this study contributes to. This is especially as UCIs continue to use newer tools like social media to rapidly amplify the spread of their narratives to large, targeted audiences—with social media also being an industry whose business model places public health in direct conflict with their profits. Transparency and accountability of sources must also be promoted to expose any potential conflicts of interest, so agendas, like PAHCF’s, can become more transparent to audiences. Strategies employed by UCIs are powerful, but also predictable as they utilize tactics from the same playbook [30]. Public health needs to be equipped to respond and effectively counter this pollution, particularly where industry and public health interests intersect.

In addition to awareness of the implications on health discourse, public health professionals and policy-makers also need to be better equipped to confront industry interference. Increasing research in the areas of how UCIs utilize campaigns and engage in political lobbying activities, especially on novel platforms such as social media, should be conducted. Impact evaluations to measure the effects UCIs can have on public policy, as well as on public perceptions, health and voting behaviours, through the use of tools such as Meta and other social media platforms, would provide evidence and inform development of policies and regulations to prevent widespread negative public health consequences. This should include the spread of mis- and disinformation; impacts on democratic values and processes; user and third-party data access and surveillance [24]; and, considering new technology’s ability to more specifically target their desired audiences, evaluating implications for affected populations. It has also been shown that UCI interference increases in response to the development and implementation of regulatory policies [71]; further analysis, evaluation, and awareness of such activities would strengthen preparedness and responses by policymakers and public health professionals to such interference.

### Limitations

Limitations to consider on the implications to public health determined by this study include that this is only one campaign of many, on one social media platform amongst others. Meta also does not publicly provide number of impressions for all advertisements; data scraping was required to collect this information, and researchers are limited by the information companies are willing to provide. While demographic data was collected, such as matched gender and age metadata with advertisements impressions, time constraints of this project prevented examination to be within the scope of this analysis; this will be examined in future research. Further, a large number of specific claims were made in advertisements (such as specific increases in income taxes or numbers of rural hospitals under threat of closure), and it was beyond the scope of this project to examine the validity of each claim. Influence on voting behaviour could not directly be determined from this study, however, further study should be conducted to measure these effects as legislation does or does not progress in the implementation of UHC policies in the US.

## Conclusion

Like other campaigns led by UCIs, this $1 million USD digital campaign is well-funded and designed to specifically (and more effectively) target different demographics of users to increase doubt in the benefits of UHC, undermine public trust in government and evidence, and promote public alignment with their own messaging and their preferred solutions. In order to effectively counter and pre-empt health-harming UCI tactics, public health professionals need to gain a better understanding of the strategies UCIs utilize to deflect attention away from their underlying health-harming intentions, how they evolve in response to changing threats and context, and understanding how distorted views of public health policies can become so widespread, shape legislative agendas, influence health behaviours, and become resistant to change [30,72–74]. This particularly requires further exploration when these messages are being delivered through large and mostly unregulated social media platforms, which enable UCIs to exploit platform tools at the expense of public health.

It is essential that the public health community, decision-makers, health providers, researchers, and the public are better informed and prepared to prevent health-harming industries from using both traditional and novel platforms to their advantage. New research and policy initiatives in these areas would help to generate more data, analysis and measurement on how commercial determinants are linked to health outcomes, and inform development of comprehensive policy interventions. Application of frameworks such as the Commercial Determinants of Health Index would support targeting of priority interventions in CDOH research, public health, and policy [23].

## Supporting information

S1 Data Coded data [75]. (XLS)
